# Biology of *Culex sitiens*, a Predominant Mosquito in Phang Nga, Thailand after a Tsunami

**DOI:** 10.1673/031.012.1101

**Published:** 2012-01-27

**Authors:** Samrerng Prummongkol, Chotechuang Panasoponkul, Chamnarn Apiwathnasorn, Usa Lek-Uthai

**Affiliations:** ^1^ Infectious Diseases and Epidemiology Program, Faculty of Graduate Studies, Mahidol University, Bangkok, Thailand; ^2^ Department of Medical Entomology, Faculty of Tropical Medicine, Mahidol University, Bangkok, Thailand; ^3^ Department of Parasitology and Entomology, Faculty of Public health, Mahidol University, Bangkok, Thailand

**Keywords:** *Culex sitiens*; filariasis vector; biting cycle; breeding habitats; colonization; longevity

## Abstract

A tsunami affected area in Phang Nga province, Thailand was explored randomly as some freshwater sites had changed into brackish-water sites. A survey of four areas found *Culex sitiens* to be the most dominant mosquito species.This mosquito prefers to breed in putrefied water with garbage and it was found in almost every stagnant, brackish-water site in full sunlight. The larval density was more than 300 larvae/dip/250 ml water. Its biting cycle, determined by human landing catch, was nocturnal, with a single peak at 19.00–20.00 hr. The maximum rate was 108 mosquitoes per person/hour. The biology of the mosquito was studied by colonization in natural water under laboratory conditions. The mean number of eggs per raft was 158.1 ± 31.7, hatchability 96.6 ± 4.1%, development from 1st instar larvae to adult was 8.8–11.7 days, and longevity of adult males was 7.3–41.3 days and females 11.0–52.7 days. The ratio of adult males to adult females was 1:1.1 ± 0.2.

## Introduction

In 2009 a tsunami hit the Andaman Coast, which is 954 kilometers in length in the southern part. Areas almost two or three kilometers from the coastline were devastated by the power of the waves, resulting in several brackish-water sites (Wattanawaitunechai et al. 2005). These new ponds containing salt marshes are potential breeding sites for mosquito vectors. Some of the freshwater sites have changed into brackish-water sites due to being filled with seawater. Larvae of *Culex sitiens* Weidemann (Diptera: Culicidae) and *Anopheles sundaicus* Rodenwaldt have been observed a year after the disaster in every water site at moderate to high densities of 40–50 larvae/dip to >100 larvae/dip (250–300ml container). A nuisance biting mosquito species was observed as 42 mosquitoes per man per 10 minutes at 19.00 hr (Apiwatthnasorn et al 2005). This result agreed with the reports of the local people in the affected areas. Apiwatthnasorn et al. (2005) also discussed the irregular increase in the mosquito population compared with the pre-tsunami period.

Even though there has been no report of an epidemic of mosquito vector-borne diseases after the tsunami, a concern was that local Myanmar laborers, who are considered as carriers of Japanese encephalitis and filariasis, might initiate a local resurgence of disease. Human filariasis is caused by the nematodes, *Brugia malayi* Brug (Spirurida: Onchocercidae) and *Wuchereria bancrofti* and it is a public health problem in the southern part of Thailand. Filariasis is a zoonotic infection that is vectored by mosquitoes. It is endemic in Narathiwat, Nakhon Si Thammarat, Surat Thani and Krabi provinces (Phantana et al. 1978). Japanese encephalitis is caused by a virus that infects the nervous system. It is common in Thailand (Umenai et al. 1985; Solomon et al. 2000) and is transmitted from birds and pigs to humans by Culex mosquitoes. *Cx .siitens* was found to be completely refractory to experimental infection with *B. malayi.* Despite the presence of high larval and biting densities, it appears to play no role in *B. malayi* transmission in the southern part of Thailand (Prummongkol *et al.* 2009). Iyengar (1953) reported that *Cx. sitiens* showed a few filaria larvae in a very early stage of development, but the larvae showed signs of degeneration, such as distortion, swelling, and vacuolation, indicating that the infection was not likely to develop any further. *Cx. sitiens* is a potential vector for Japanese encephalitis (Vythilingam *et al.* 2002). *An. sundaicus* is a malaria vector in coastal areas of Thailand and other countries (Chowanadisai et al. 1989).

The biology of *Cx. sitiens,* a possible vector in tsunami affected areas, including their breeding habitat, biting activity was determined so that it could be investigated and monitored a new prevention and control measure strategy in the Phang Nga area.

## Materials and Methods

A survey in the post-tsunami affected areas was explored in a random fashion during February to April 2005. Some freshwater sites had changed into brackish-water sites. The denatured and polluted water sites that formed had become breeding areas for *Cx. sitiens.* The collection stations were set at Phang Nga Naval Base, Ban Keuk Kuk, Ban Bang Ka Ya and Ban Nam Khem, Phang Nga province.

### Natural breeding habitats

Surveys of breeding places of *Cx. sitiens* were carried out and mosquito species and aquatic predators were determined as well as some quality parameters of the breeding water, such as pH, conductivity, total dissolved solids, dissolved oxygen, salinity and temperature. The water quality was recorded using a Sension 156 Portable Multiparameter Meter (HACH, www.hach.com). Identification of the collected mosquitoes was based on adults emerging from the pupae.

### Biting cycle

Collection stations were established at all study sites to determine the biting cycle of the mosquito studies. The biting cycles were conducted outdoors between 1800–2400 hours, by human landing catch. Mosquito counts were made hourly for 45 minutes by two groups, each with two persons, for three periods of time during 3 consecutive nights from Nov. to Dec. 2005. All mosquitoes were identified as to species by using a standard dissecting microscope and taxonomic keys (Bram 1967; Ratanarithikul *et al.* 2005). Female *Cx. sitiens* mosquitoes were transferred into paper cups with screen tops (approximately 50 mosquitoes per cup) and cotton wool soaked with 10% sugar solution was offered as a food. These cups were placed in a cooler covered with a wet towel. Ice packing was used to keep them cool and provide humidity during transportation.

**Table 1.**
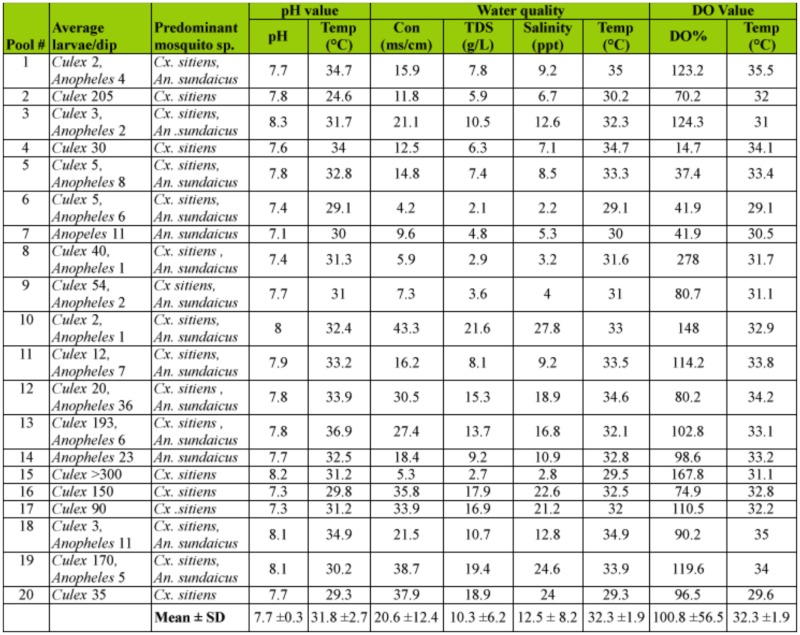
Larval survey and water quality in the observation breeding places in the tsunami affected area, Phang Nga Province, Nov–Dec 2005.

### Biology Laboratory colonization of *Culex sitiens*

The mosquitoes were reared individually to get single colonies using a modified procedure (Panicker *et al.* 1981; Limsuwan *et al.* 1987). After returning to the laboratory, the female mosquitoes were released into a 30×30×30 cm cage as starting colonies and the females were given a blood meal from a golden hamster by placing it inside a mosquito cage overnight. The engorged females were transferred into paper cups (approximately 15 individuals per cup) and cotton wool soaked with 10% sugar solution was offered as food. About three or four days after feeding, each mosquito was transferred to a plastic cup containing 15 ml natural water which was carried from the field at the collecting areas for oviposition. Egg rafts were separated individually in plastic cups with the same natural water to observe hatchability. On the following day, the number of eggs that hatched were scored from 20 egg rafts. The number of eggs in each raft, the duration of different larvae instars, pupae and adults recorded every day. The larvae were reared in a plastic trays with about 1000 ml of natural water (from field study area) per tray. A mixture of Powered Fish 2000 (White Crane Aquarium Co. Ltd. (www.whitecranev88.com) and water was provided as larval food. Food was added to each plastic rearing tray at each of the 4 instars, 0.5, 1.0, 1.5 and 2.0 ml respectively. Numbers of male and female mosquitoes that emerged were counted and recorded, cotton wool soaked with 10% sugar solution was provided as food for the adults. The insectarium used was not air-conditioned, the temperature and relative humidity were recorded and measured. The rearing temperature was 30.8 ± 0.5^°^C (range 29–31^°^C) with approximately 68.2 ± 3.1% (64–70%) relative humidity.

### Observations on longevity

The longevity of eggs, larvae, pupae and adults was assessed. Thirty of each of the newly emerged males and females were kept separate in paper cups covered with screen tops, with cotton wool soaked with 10% sugar solution provided as food. These sugar pads were provided regularly and changed every day until the adults died. Paper cups were checked daily and dead mosquitoes were counted and removed until all of mosquitoes died. The sex ratio of the adult male and female was also determined.

## Results and Discussion

### Nature characteristics of breeding habitats

Mosquito larvae were collected from breeding habitats in the tsunami-affected areas of Phang Nga province, from Nov. to Dec. 2005 and the results are shown in Table 1. The immature stages of *Culex sitiens* inhabit sunlit stagnant water, including small pits, pit lakes, wells, swamps, ponds, canals, particular shrimp ponds and water that collects in abandoned tin mines, as in previous studies (Apiwathnasorn *et al.* 2005). Their breeding habitats varied in size from small pits (1×2 m2) to large water reservoirs such as pit lakes or water collections in abandoned tin mines (220×730 m2). The water may be contaminated with garbage, floating pieces of dried leaves, filamentous green algae (*Enteromorpha* sp.) and grasses. In some breeding places fishes, naiads and shrimp were found. It was found that almost all sites of breeding places were positive for *Cx. sitiens* larvae with a maximum larval density of more than 300 larvae per dip (water volume 250 ml). The water quality of breeding habitats is shown in Table 1. The mean salinity was 12.5 ± 8.2 ppt.

Three larvae mosquito species were found with breeding habitats in tsunami affected areas, Phang Nga province. *Cx. sitiens* have been described as a brackish-water breeding mosquito and are generally regarded as being restricted to costal regions (Bram 1967; Bick 1951; Colless 1957c). In our study we found that *Cx. sitiens* preferred to breed in more putrefied water with garbage as previously reported (Apiwathnasorn *et al.* 2005). *Cx. sitiens* was the most dominant species, but *An. sundaicus* and *Aedeomyia catasticta* Knab were also recorded.

### Biting activity of mosquito

**Figure 1.**
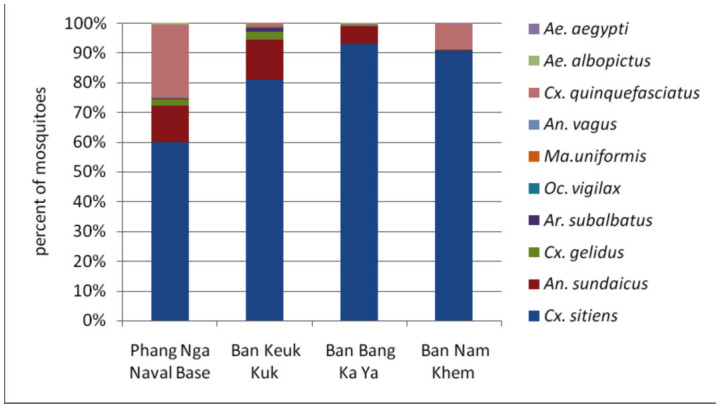
Percentage of mosquito species by human landing catch Nov–Dec 2005. High quality figures are available online.

The biting cycle of *Cx. sitiens* was determined by human landing catch. There were 10 mosquito species collected from 4 stations mentioned above during the biting cycle study. These were *Cx. sitiens, Cx. gelidus, Cx. quinquefasciatus, Mansonia uniformis, An. sundaicus, An. vagus, Aedes albopictus, Ae. aegypti, Ochlerotatus vigilax,* and *Armageres subalbatus. Cx. sitiens* was the most abundant species collected from 4 stations. A total of 5,095 mosquitoes were collected by human landing catches. The most abundant species was *Cx. sitiens* 1,434 (81.1%), *An. sundaicus* 451 (8.9%), *Cx. quinquefasciatus* 367 (7.2%), *Cx. gelidus* 78 (1.5%), *Ar. subalbatus* 27 (0.5%), *Oc. vigilax* 11 (0.2%), *An. vagus* and *Ma. uniformis* 9 (0.2%), *Ae. albopictus* 8 (0.2%) and *Ae. aegypti* 1 (0%) (Figure 1).

*Cx. sitiens* has been reported in Thailand and in neighboring countries including India, Indonesia, Malaysia, Vietnam, Myanmar and Cambodia (Chowanadisai *et al.* 1989; Harinasuta 1974; Chow 1970). It was previously regarded as a secondary vector of malaria in Thailand (Chowanadisai *et al.* 1989; Chow 1970; Rattanarithikul and Panthusiri 1994). *Cx. quinquefasciatus* is the vector of *W. bancrofti* urban type (Burke and Leake 1988). *Cx. gelidus* is the vector of Japanese encephalitis (Burke and Leake 1988). *Ar. subalbatus* is a potential vector of *B. pahangi* in experimental studies in Thailand and Malaysia (Sucharit and Sucharit 1988). *Oc. vigilax* appears to be the principal vector of non-periodic filariasis in New Caledonia (Iyengar 1954) and has been found naturally infected with Murray Valley encephalitis virus in Australia (Doherty *et al.* 1963). *Ma. uniformis* was incriminated as a vector of brugian filariasis (Harinasuta *et al.* 1970). The susceptibility of *An. vagus* to nocturnally subperiodic *B. malayi* was studied in the laboratory and the results showed that the infective rate was only 0.74% because they have well developed cibarial armatures that damage 94.57% of ingested microfilaria (Choochote *et al.* 1987).

**Figure 2.**
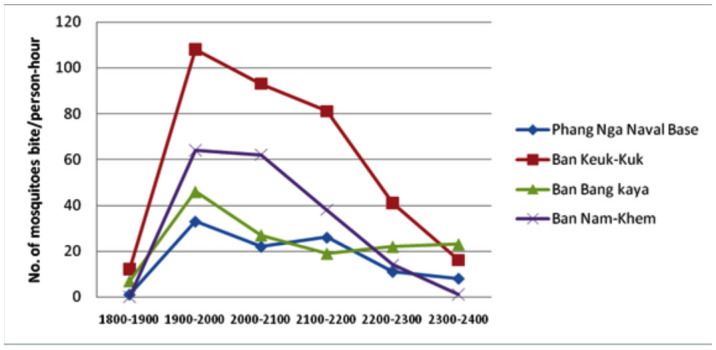
Overall biting activity of *Culex sitiens* mosquitoes at 1800–2400 hr, combining biting cycles from 4 collecting stations Nov–Dec 2005. High quality figures are available online.

The biting cycle of *Cx. sitiens* mosquito which was determined by human landing catch was nocturnal, with a single peak at 19.00–20.00 hr (Figure 2). The maximum biting rate of *Cx. sitiens* was 108 mosquitoes per person-hour, similar to the result from Apiwathnasorn *et al.* (2005), when 135 mosquitoes per person-hour were reported. Their biting densities were very high at 108, 64, 46, and 33 bites per person-hour at peak periods in Ban Keuk Kuk, Ban Nam Khem, Ban Bang Ka Ya, and Phang Nga Naval Base, respectively.

### Mosquito colonization

Each raft of eggs was hatched in natural water from the breeding habitat, which had a pH 7.4 (temperature 32.1°C), conductivity 5.7ms/cm, total dissolved solid 2.9g/l, salinity 3.1ppt (32.3^°^C) and dissolved oxygen 60.4% (32.6^°^C). Based on the ecology and biology of *Cx .sitiens* obtained from this study, it was successfully colonized and could be consecutively colonized in insectariums at a temperature of 30.8 ± 0.5^°^C (range 29–31^°^C) and 68.2 ± 3.1% (range 64–70%) relative humidity.

Observations of the number of egg per raft, hatchability, immature survival and sex ratio were made. The mean number of eggs in each raft was 158.1 ± 31.7 (range 104–223). The egg stage lasted for 2–3 days and the larvae emerged through a transverse slit at the anterior end of the egg. Among deposited egg rafts, all of them were viable with average hatchability 96.6 ± 4.1% (range 82.3–100%). The life duration of different larval instars and pupa was studied by measuring immature survival from 1st instar to pupa stage and was 92.2 ± 12.1% (range 55.3–100%). The ratio of adult males to adult females was 1:1.1 ± 0.2.

The duration of different larval instars, pupa and adult were studied and the results showed that the mean range of duration of the first, second, third and fourth larval instars were 3.2 ± 1.6, 5.2 ± 2.2, 6.6 ± 1.4 and 7.6 ± 1.4 days, respectively. Then at 8.9 ± 1.6 days they became pupae and emerged as adults at 10.3 ± 1.4 days.

Observations on the duration of thirty adult males and females determined in laboratory rearing are shown in Table 2. Three trials of longevity were performed. Male *Cx. sitiens* fed 10% sugar solution, survived for 7.3–41.3 days, while females survived for 11.0–52.7 days.

**Table 2.**
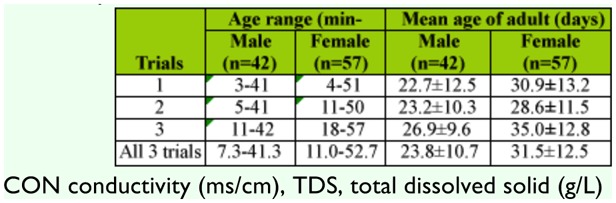
Longevity of adult males and females *Culex sitiens* under laboratory conditions.

The ratio of adult males to adult females was 1:1.1 ± 0.2. The duration of different larval instars, pupa and adult were studied and the results showed that the mean range of duration of the first, second, third and fourth larval instars are 3.2 ± 1.6, 5.2 ± 2.2, 6.6 ± 1.4 and 7.6 ± 1.4 days, respectively. Then at 8.9 ± 1.6 days they became pupae and emerged as adults at 10.3 ± 1.4 days.

## Conclusion

A large-scale environmental change has been observed post-tsunami period in tsunami affected areas of Phang Nga province. The ecology and biology of *Cx. sitiens;* such as their breeding habitat and biting activity were studied, and there were increasing numbers of *Cx. sitiens. Cx. sitiens* was the dominant mosquito species. The maximum biting rate was 108 mosquitoes per person-hour. It has been described as a brackish-water breeding mosquito but it was found that they preferred to breed in more putrefied water with garbage. The duration of different larval instars, pupa and adult were studied and the results showed that the mean of duration from larvae to adult was about 11 days.
